# Regenerating physiotherapy curriculum in higher education: diving into planetary health and service-learning conceptual synergies

**DOI:** 10.3389/fpubh.2025.1556869

**Published:** 2025-07-11

**Authors:** Berta Paz-Lourido, Álvaro Ribeiro-Chaves

**Affiliations:** ^1^Innovation Unit in Sustainable Development, Health and Global Justice Through Service-Learning, Department of Nursing and Physiotherapy, Institute for Educational Research and Innovation, Balearic Islands Health Research Institute Foundation, University of the Balearic Islands, Palma, Spain; ^2^Innovation Unit in Sustainable Development, Health and Global Justice Through Service-Learning, University of the Balearic Islands, Palma, Spain

**Keywords:** planetary health, physiotherapy, service-learning, higher education, curriculum

## Abstract

The physiotherapy curriculum has been modified over the years to address patients, community and health care system needs more fittingly. Recent attention is being paid to the need for an eco-social transition of the profession. This article delves into conceptual frameworks that underpin the urgent need to regenerate the curriculum, fostering a vision of humanity and the planet as deeply interconnected and indivisible. This implies not only becoming aware of the environmental impact of educational and clinical physiotherapy practices but also to reframe education through the lenses of planetary health. This calls for active engagement, a solution-oriented approach to education, and a reimagining of humanity’s relationship with the planet. Service-learning operates as an experiential critical pedagogy, blending hands-on engagement with reflective analysis to challenge students’ assumptions and foster transformative learning rooted in social justice. This form of experiential critical pedagogy empowers students to learn while serving their communities in a range of areas—most notably in response to the urgent challenges of planetary health. By aligning planetary health with service-learning—an action-based pedagogy that cultivates student empowerment and civic responsibility—we can envision a regenerative transformation of the physiotherapy curriculum. This work’s aim is to gain epistemological insight on the social-ecological complexity on this matter. The article discusses four rationales that permeate a framework for this regenerating purpose in the light of the Global Action Plan on climate change and health. It tackles planetary health, higher education, physiotherapy, and service-learning as a pedagogy orientated to action and critical reflection. All this opens the gate to rethink the role of physiotherapy education in the Anthropocene. Higher education needs to interconnect individuals, communities, and the natural world, promote shared governance and democracy, stimulate critical thinking and embrace diversity. Education in physiotherapy needs to underscore cognitions and academic proficiency, emotional intelligence, social consciousness, and ethical awareness, matchmaking learning experiences with local and global challenges. There is a need to advocate for pedagogical approaches that inspire hope and prepare students to contribute meaningfully to their communities, upholding principles of equity and intergenerational justice.

## 1 Introduction

For decades, physiotherapy’s educational curriculum was aimed at training professionals to meet various individual needs linked to movement and functionality mainly focused on the specific competences (1, 2). Subsequently, the professional profile evolved, focusing on its potential to promote the health of the population from a public and community health perspective, understanding the social determinants of health, promoting interdisciplinary research, leadership and advocacy roles (3). This led to the need to expand the scope of the physiotherapy curriculum giving emphasis to transversal competences (4).

Over the last years, the biomedical perspective has permeated education and clinical practice, so that the evidence based on dominant research has also raised a growing concern about the loss of humanistic value and the lack of critical theoretical reflection in the profession (5). This evolution is not unique to physiotherapy and other health professions are going through a similar process reframing the curriculum to meet urgent global challenges. Currently, an eco-social transition of the profession is taking place, questioning the physiotherapy narratives that separate the foundations of the discipline from the Indigenous tradition, the uses of nature not as a resource but as constituent of the intrinsic good of the profession, and the participation of physiotherapists in the construction of more inclusive, supportive and environmentally responsible policies (6). Worldwide events such as the COVID-19 pandemic put the focus on physiotherapy as a crucial therapeutic strategy but also opened the discussion regarding the lack of consideration of the profession in international debates on how pandemics and chronic diseases are effects of the lack diversity, deforestation or pollution (7–9). The One Health initiative posed by the World Health Organization is only possible if there is a change in the way of understanding the health professions (including physiotherapy) and with it, the educational process for students to acquire the necessary skills. As will be seen later, conceptually One Health and Planetary Heath are not the same, but both require competencies from physiotherapists different from those traditionally accepted as health practitioners. The physiotherapy profession has a growing interest and engagement in addressing climate change and its effects on health (10).

Ensuring equitable human development is important, but such development should be accomplished without further destabilizing the Earth’s natural systems (11). As this is perhaps the greatest challenge of our time, people worldwide are increasingly facing the pressing challenges of today’s interconnected environmental, social, and health crises. Academic and scientific personnel call for greater biodiversity and human health protection by preserving existing forests and improving the management of new or expanding ones (12). One way of navigating these seemingly opposing objectives is by detailing an understanding of the trade-offs and synergies between human progress and the development and environmental targets (13). That means designing a path to the future that offers sustainability and regeneration of the Earth’s system, in which nature’s benefits, risks, and responsibilities are shared fairly across the world (14). To transition into environmentally responsible and socially inclusive physiotherapy-care systems, universities should embed planetary health education and training across higher education curricula (15). It is at this point where experiential pedagogies such as service-learning have a great potential to change the positivistic university inertias driven by institutional prestige and others, with impact in physiotherapy education (16). Moving away from predictive higher education, this work places an epistemological insight on the social-ecological complexity, valuing the expectations of each actor in physiotherapy education.

The article dives into concepts that could support a process of regeneration of physiotherapy curricula toward planetary health, based on a critical review and synthesis of existing literature, proposing innovative pathways for higher education. This implies not so much incorporating content on what planetary health is or is not, but how this can be embedded transversely and longitudinally into the curriculum in a normalized way, not as a fad. Studies on service-learning and planetary health are still very incipient, and inexistent (to the authors’ knowledge) in their relationship with physiotherapy. So, this work also aims to contribute to the development of a comprehensive approach to how higher education and its surrounding communities can foster a regeneration of physiotherapy programs considering the WHO’s Global Action Plan on Climate Change and Health (17).

The question that underlies this work is what rationales can be considered for the regeneration of the physiotherapy curriculum, how can they be articulated and what dimensions can emerge from that process. In this case, the pedagogical alignment includes four rationales: planetary health, regeneration of higher education, service-learning, and physiotherapy in order to propose a synergic path toward the regeneration of physiotherapy education.

## 2 The rationale for planetary health

Climate change poses a substantial threat to human health, leading to millions of additional deaths and illnesses due to increasingly striking extreme weather events (18). It especially affects the populations of low and middle-income countries (LMICs), owing to scarce resources, inadequate planning, and insufficient investment in health care, environment, and related sectors (19). Climate change is associated with negative health outcomes, increasing mortality and exacerbating cardiovascular and respiratory illness, mental health disorders, and other climate-sensitive health outcomes (20). These examples demonstrate the causal pathways that link the three global pressures of climate change, biodiversity loss, and infectious disease (21). This has attracted the attention of the different health professions, initially in its scientific performance but also in its educational, political advocacy and clinical roles.

Conceptual elements of planetary health have been discussed in the last years expanding on previous public health considerations (22). The Kuala Lumpur call to action (23) defines planetary health as a global movement, analytical framework and field of work what requires a solution orientated approach to promote a resilient and regenerative future for all. Institutions, educators, and learners that incorporate this framework would need to shift away from a business-as-usual, siloed approach to education (24). The São Paolo Declaration on Planetary Health (25) states recommendations for health practitioners, health sector, researchers and university to advance in the so-called Great Transition. This transition to a sustainable future requires reconciling the respect for the Earth limits with the justice principles reframing considerations as prosperity and progress (26). For institutions to adapt planetary health as a guiding framework for an institution-wide approach, the issue of contextualization, transdisciplinarity, epistemological diversity, solution-oriented, action-based, and transformative approaches to education should be intentionally designed through strategic, logistical, and resource lenses (27, 28). All this would require inclusive relationships, thoughtful strategy, effective communication, and transformational partnerships (29).

Planetary health is distinct from One Health (30). While the first emphasizes the function of climate change, biodiversity loss, and environmental degradation on human health (31), One Health promotes integrative collaboration among multiple disciplines (32), reinforcing the disease control and global health security. While planetary health calls for systems thinking and complex interactions between ecological, social, and economic systems concerning human and ecosystem health to address global health challenges (33), the second emphasizes integrated surveillance, research, and policy to address shared health threats between humans and animals (34). In terms of resemblances, both recognize the interdependence of human, animal, and environmental health, emphasizing ecological interactions. Planetary health aligns with One Health in acknowledging the importance of ecosystems, climate change, and sustainability, and vice versa occurs in acknowledging the interconnectedness of health and ecosystems (35, 36). Both are highly complementary approaches based on transdisciplinary, multisectoral, and system-based approaches to health, but challenges remain when translating ideas into policy and practice (37). One Health and Planetary Health contribute to building a much stronger research community to collectively address public and global health challenges in an integrated way (38) pushing public health to evolve (39).

In any case, if we consider the frameworks used by the World Health Organization for the operationalization of the Global Action Plan on Climate Change and Health (17) one observes that four approaches are considered: (1) Sustainable Development Goals, (2) Health in All Policies, (3) One Health and (4) Planetary Health. These frameworks contribute to define the 15 key principles of this plan: Adaptability, Holistic approaches and collaboration, Community orientation, Human rights, Environmental justice, Innovation, creativity and technology-based, Evidence-based practice, Multisectoral partnerships, Financial efficiency, Social determinants of health, Gender equality/Gender inequalities and differences in needs and opportunities, Traditional and indigenous knowledge, Local and regionally led strategies, Vulnerable populations and Health equity. This plan brings implications for higher education and, therefore, for physiotherapy education.

## 3 The rationale for regenerating higher education

Regenerating physiotherapy curriculum is not possible without being framed in a broad higher education transformation. Higher education includes universities, universities of applied sciences and other forms of education considered to be the highest level of education in a given country. Its constitution has evolved since the first universities established in the Middle Ages in Europe and so has its purpose (40). According to the United Nations Educational, Scientific and Cultural Organization (41), higher education is a rich cultural and scientific asset that favors personal development and economic, technological and social transformations. It also stimulates the exchange of knowledge, research and innovation, and equips students with the necessary skills to respond to the constant evolution of the labor market ensuring them economic security. But UNESCO (42) has claimed in the last years for renovated structures and procedures to ensure real transformations such as increasing its interdisciplinarity by focusing on and promoting the critical and active spirit of citizens, as this would contribute to sustainable development, peace, well-being and human rights.

Higher education plays a vital role in training future professionals to mitigate climate change and propagate social and governance measures to foster sustainability (43, 44). Unlike previous agendas, such as the Millennium Development Goals and Education for All, the Sustainable Development Goals, which make up the 2030 Agenda for Sustainable Development, explicitly refer to higher education as part of the vision of lifelong learning for all (45). Higher education is urgently required for sustainable development, innovating not only for individual impact in the classroom, but also to advance institutional change and decisively influence the teaching and learning discourse of higher education (46).

In terms of planetary health, contemporary universities could address diverse aspects of the problem. On the one hand, they must become carbon-neutral institutions by adopting low-carbon operating practices, and on the other hand, they must develop curricula and pedagogical approaches to educate students (and, by extension, communities) about the imperatives of climate change mitigation and adaptation. In addition, universities, as trusted organizations, must increase the evidence of the impact that different policies have to generate large-scale but context-sensitive changes (47). But since planetary health considers systematic feedback loops for causality (98), the same approach could be applied to higher education institutions: their actions influence the environment and the community, but also vice versa. Therefore, the role of higher education within the Global Action Plan on Climate Change and Health (17) could be also addressed by enhancing the so-called “third mission” of universities through community participation.

All the above bring several scenarios to the discussion. From the perspective of ecological justice (that is, with nature), the embedding of higher education with planetary health topics, would engage in more sensible discussions on climate change, addressing the disparities between official international climate policy and societal and ecosystem needs (48). Higher education would advocate strategies that could widen the investigation on local and regional political ecology (49). From the perspective of social justice, higher education would manage solidarity, collaborative and empowering relationships that could reduce social-economic inequality, social discrimination and oppression worldwide (50). From the perspective of cognitive justice, higher education would foster dialog between science and other understandings, which are essential to equality between inclusion and participation (51). Through the concept of learning together, higher education would engage in an ongoing reflexive praxis that would generate knowledge for participants within their communities while also generating knowledge about how we learn. From the perspective of historical colonialism justice, higher education would advocate broader recognition of how socially and regionally uneven concentrations of wealth, which have resulted in climate-changing emissions, were created in the first place (52). From the perspective of gender justice, higher education would recognize that gendered vulnerabilities require tailored adaptation measures (53).

For higher education to be able to effectively cope with these challenges and future scenarios, pedagogies that actively engage people in learning and behavioral change toward actions for sustainability will be required (54). Regenerative education is an emerging strand of educational theory and practice based on the way in which human communities and individuals relate to themselves and the environment (55, 56). That means valuing improved social-ecological systems for the purpose of mutually flourishing. The proposal for the regeneration of higher education implies the need for a far-reaching transformation, which pays attention to lived experiences, interpretation and co-construction of reality. From a socio-critical point of view, a real transformation of educational institutions requires educators to be more aware of their role, which can be enhanced through self-reflective and dialogic processes (25). Transdisciplinary research, teaching and learning will be necessary and possible by promoting a solution-oriented praxis based on epistemological diversity (57).

Regenerating education means enabling individuals to tackle complex and emotional topics related to planetary health without feeling overwhelmed. This would imply redesigning learning institutions by embedding climate change types of justice and planetary health education in curricula, across all levels, disciplines, sectors (geographies/indigenous/spiritual) and pedagogies (58). Technology would be used in each setting increasing adequate accessibility to resources for planetary health and civic engagement (59). Environmental health curriculum and communication skills would effectively advocate for climate leadership empowerment and workforce preparedness (60). The energy, awareness and ambition of youth would complement the authority and expertise of subject matter experts if the educational barriers to youth engagement in high-level decision-making are removed (61). That requires equipping and enabling learners to drive transdisciplinary and mutually reinforcing actions.

## 4 The rationale of physiotherapy

The Policy Statement of Climate Change and Health states that World Physiotherapy recognizes the existence of a climate change emergency as the greatest threat to human health in the 21st century (62). This statement should not be seen only as a starting point but as a further step in the expected growing interest of physiotherapy in recognizing roots in the profession that combine environmental awareness, clinical reflection and ethical thinking within a sustainability and patient safety context. It aligns with the International Classification of Functioning, Disability and Health (99) and recognizes the important role of the environment in people’s functioning, ranging from physical factors (such as climate, terrain or building design) to social factors (such as attitudes, institutions, and laws). It also links with the ethical principles for individuals and physiotherapy organizations (63), since the health of people and communities is not disconnected from the environment. Previous works have claimed that there is a strong global call for the physiotherapy profession to take action to advocate for sustainable practice at individual and organizational spheres (10).

It has been stated that physiotherapists are at the forefront of tackling diseases and supporting people with disability and could advocate for practices addressed to sustainability and health promotion (6). Nature prescriptions have been integrated within face-to-face therapies offered by physiotherapists, with emerging research highlighting nature’s role as a non-pharmaceutical means of supporting rehabilitation (64). In interventions involving people with long-term health conditions spending time in parks, gardens, forest/woodlands and wetlands, improvements were reported in the participants’ psychological, physical and social well-being (65). Harnessing nature can lay the foundations for more health equity and sustained behavioral change that extends beyond the clinical environment to everyday life and routines, which might not otherwise be achieved in tightly controlled indoor settings (66).

Embedding planetary health within physiotherapy education would support Indigenous and traditional knowledge, new voices to inform physiotherapy healthcare practice (67). It would discuss how promoting environmental sustainability in physiotherapy health care could support progress on social determinants of health, health equity, and respect for cultural diversity. It would favor the discussion of how physiotherapy health professionals should contribute to mitigation, adaptation, advocacy, and activism regarding sustainable development, and environmental stewardship (58). The redesign of urban areas toward health-promoting spaces, reducing pollution, increasing walkability, wilderness and green spaces through shared governance models would be a normalized area for physiotherapy education in health promotion and public health, since these are also possibilities for concrete nature-based actions with a local and global impact on planetary health (68).

In this sense, it is necessary to take into account existing initiatives in higher education such as the International Network of Health Promoting Campuses or the International Network of Sustainable Campuses, on which physiotherapy education can not only pivot but also propose innovative experiences. In relation to local communities, pedagogical approaches such as service-learning increase reciprocal relationships between academic and community partners. Considering the large number of organizations whose mission is aligned with environment, sustainability, climate change, and planetary health, service-learning could permeate physiotherapy faculties and departments in a more agile and effective way than other teaching methods.

## 5 The rationale of service-learning

Service-learning in higher education is an experiential educational pedagogy in which students engage in community service, reflect critically on this experience, and learn from it personally, socially and academically. The activities address human, social and environmental needs from the perspective of social justice and sustainable development, and aim at enriching learning in higher education, fostering civic responsibility and strengthening communities. It brings together students, academics and the community whereby all become teaching resources, problem solvers and partners. In addition to enhancing academic and real-world learning, the overall purpose is to instill in students a sense of civic engagement and responsibility and work toward positive social change within society. UNESCO (25) states that *“service-learning and community engagement soften the walls between classes and the community, challenge students’ assumptions, and connect them to systems, processes, and experiences beyond their own experiences*.” Although there are diverse considerations about the relevance of this pedagogy in higher education, the core principles are clear. An authentic service-learning experience is designed in a way that engages students in learning experiences by addressing real needs through partnerships with community entities. In turn, community service activities are addressed to enhance students’ learning. This “service” includes actions as direct service, indirect service, advocacy and research, which implies that higher education students (all levels) can develop actions ranging from direct interventions in the community to research-based advocacy reporting. So, the distinctive character of service-learning resides in the fact that students are focused on ensuring mutual benefits for themselves and the community, promoting otherness as a value, that is, encouraging recognition of difference and diversity, both in the personal and social spheres.

Critical reflection is essential and aimed to deepen student’ learning but also community-campus partnerships (69) This is why service-learning should not be confused with volunteering, internships, or fieldwork. Service-learning is a structured and balanced pedagogical approach that integrates academic learning with meaningful community engagement (100), its roots are experiential pedagogies based on “learning by doing” (70).

Prior studies show that service-learning can act as a catalyst for activating education for sustainable development in higher education (71). This is particularly important because there are significant psychological barriers that limit individual action in terms of climate change, including a lack of knowledge and feelings of uncertainty and disempowerment (72). Experiences include data collection as a part of service-learning partnerships applied as a tool to integrate climate change and sustainability concepts in the classroom (73). Service-learning has been used to provide students with an opportunity to directly engage with organizations grappling with climate change through the collection of data, the creation of models, and the application of those models to scenarios of climate change (74). Therefore, service-learning promotes students’ ability to apply their newly acquired skills and knowledge to support partner organizations in understanding how to face and respond to climate change more clearly.

From the perspective of the faculty, students gain from service-learning experiences a deeper understanding of the impact of climate change on their discipline by allowing them to conduct real-world, applied work (75). Service-learning partnerships between students and scientists should be considered an important new avenue for updating education and practice, but also for promoting community science and planetary health advocacy. Finally, when referring to service-learning consideration should be given to the tendency of projects to evolve from simple experiences to complex, dynamic and long-term sustained collaborative projects based on strong partnerships. To facilitate this transition, the so-called “institutionalization of service-learning” allows the faculty and the university to be committed to this pedagogical approach in the long term, which also facilitates measuring its impact (76–79).

## 6 Discussing and proposing conceptual dimensions to regenerate physiotherapy higher education curricula

Contemporary higher education is called to align its purpose toward the Global Plan of Action on Climate Change and Health (17). This plan is organized into three primary action areas: (PAA1) leadership, coordination and advocacy; (PAA2) evidence and monitoring; and (PAA3) country-level action and capacity-building. Each primary area is framed in a global target divided into objectives with specific actions addressed to members states, the WHO secretariat and stakeholders. To articulate the path of this discussion only one objective of each primary action areas will be selected, although all of them are interconnected. The Objective 1. C refers to *“Empower, inform and effectively engage the health community to support climate and health action*,” the Objective 2. A is to “*Strengthen the scientific and traditional knowledge evidence base through scientifically-sound research and empirical evidence on the connections between climate change, climate action and health,”* and finally, the Objective 3. E is to “*Implement climate change and health interventions to increase climate resilience and reduce greenhouse gas emissions of health systems and facilities.”* In this sense, physiotherapy education will need to prepare future professionals to pursue these objectives and to come up with strategies for climate change mitigation and adaptation as part of their social role (80). There are six fundamental reasons to regenerate physiotherapy curricula. First, it would influence the design of transformative educational strategies that would integrate diverse ways of knowing and co-benefits for people, communities and the planet, as knowledge and spiritual traditions (81). Second, it would foster a social and ecological approach to health promotion and disease prevention and control, ranging from individual to population-level determinants of human, animal, and ecosystem health, and its underlying and mediating factors (82). Third, it would characterize the linkages between environmental changes and human health at different geospatial and temporal scales and would incorporate characteristics of complex adaptive systems (83). Fourth, it would promote equity and justice in the rights of humans and the rights of nature, giving all human populations and ecosystems, present and future, the opportunity to attain their full vitality (84). Fifth, systemic disparities would be reduced so that no population carries disproportionate burdens of environmental and health impacts while others can thrive (85). And sixth, education processes would acknowledge the structural inequities and historical and political injustices (83).

Service-learning emerges here as an experiential pedagogy, a useful approach to address new narratives regarding planetary health and climate change in higher education (54, 86, 87). As sustainability is a complex and sometimes vague idea (88), service-learning projects can intersect climate change and physiotherapy, acting as a concrete pathway for physiotherapy students to learn about and engage in their field of study. By integrating ecological awareness in service-learning projects through synchronicity with the planet’s natural cycles and sources of life (water, air, earth, and sunlight), students would consider the present and future impact their thoughts and actions have on others and place (89). That would build a sense of reverence for themselves and their community, a sense of belonging that fosters complex relationships over time, for which intergenerational service-learning projects may be adequate. Planetary health sees women as keepers of cultural identity and caretakers of the natural environment, as vessels for strong communities and networks, bonding people, place and community. Service-learning in planetary health seeks to enhance collective vitality and well-being, reinforcing human relationships with the ecosystem. Students would learn to reconstruct the collective awareness of the interconnectedness that exists within nature and re-gain ecologically bound cultural identity, pairing human relationship with their inner self and as part of nature systems. At a practical level, including nature-based physiotherapy practices in the curriculum would pose students closer to understanding the meaning of interconnectedness with nature, fostering comprehension on biological diversity recognition or eco-sustainable traditional practices. Service-learning projects can be developed to address local and global conservation goals, preserve native cultures and keep and communicate traditional knowledge, culture and sovereignty related to biodiversity preservation.

The regeneration of physiotherapy education via planetary health and service-learning means that higher education educators should make physiotherapy students more than observers, replicators and reproducers in the teaching-learning process. They should encourage students to actively participate in the “physical” world, opening the doors and windows of classrooms in their metaphorical and real sense. The description of the events linked to planetary health should go hand in hand with the self-reflection and interpretation since education is a socially constructed process. They can get inspiration from the fact that indigenous or traditional cosmologies position humans as inseparable from the Earth and all its inhabitants (58, 89). A greater connection with the rural world through community campus partnerships can also be a good strategy to establish better connections with ways of life closer to the rhythms of nature, including the experience of its unpredictability. Such an approach sets the stage for complex thinking, non-linearity, transdisciplinarity and resilience. It provides a substantial theoretical basis for the inseparable understanding of physiotherapy as a phenomenon of research, practice and education, whose multidimensional nature can be fed back through pedagogies that stimulate co-creation instead of reproduction.

**Figure 1:**
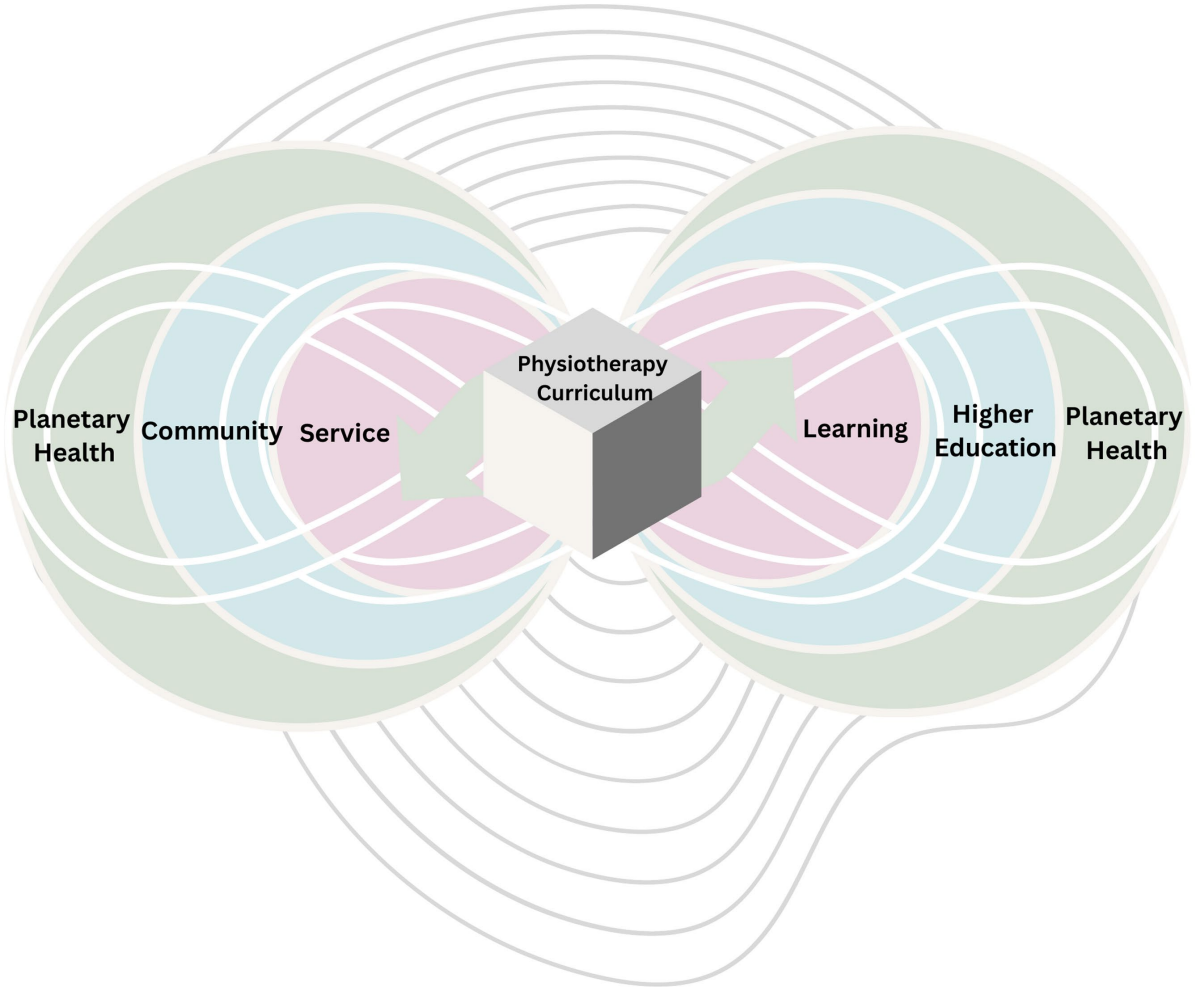
Systematic feedback loops in planetary health, physiotherapy and service-learning.

The application of the principle of inseparability implies that higher education physiotherapy curricula should embrace the fundamentals of planetary health not as a new fad or imposition but as a possibility of regeneration. This would make physiotherapy education more contextual, holistic, symbolic and relational, not limited by time. Since planetary health considers systematic feedback loops for causality this approach needs to be embedded in community-campus partnerships, for which service-learning is an appropriate pedagogical approach (Figure 1).

**Figure 2:**
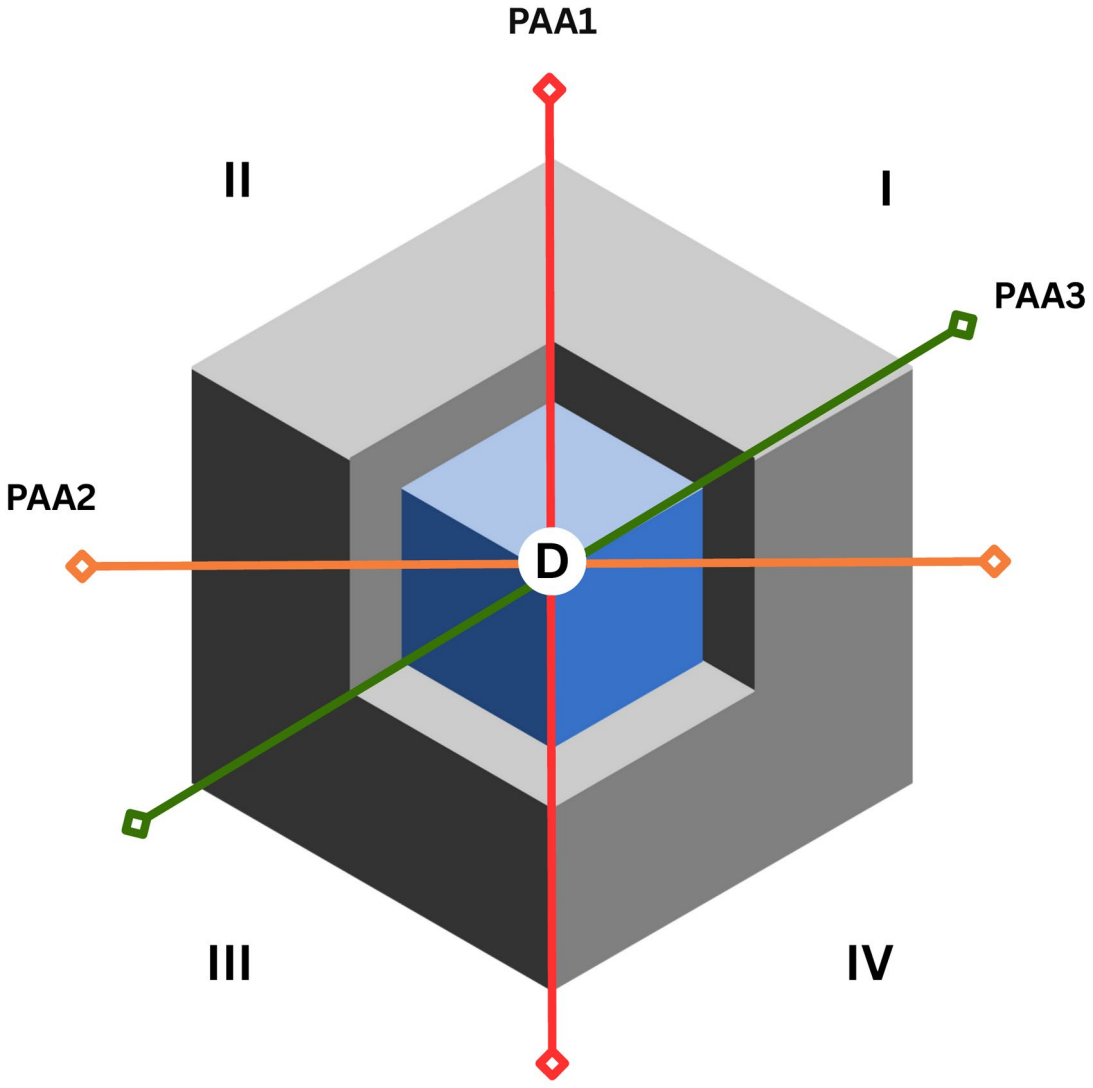
Climate change, primary action areas, domains and curriculum.

The complex notions of responsibility that involve service-learning through its co-created approach, means that students would learn and implement professional codes of conduct for maintaining a peaceful, thriving, and cooperative society grounded in love and reciprocity. The embedding of planetary health on physiotherapy curricula through service-learning can be also a way of relating the rights and needs of people, of teaching the art of governing through negotiated instruments, and promoting otherness without compromising self-determination and dignity. Due to the nature of critical reflection involved in service-learning, gender inequalities or colonialism are among the issues to be addressed (90). Therefore, incorporating planetary health through service-learning into physiotherapy higher education curricula would require consideration of four domains (Figure 2). Domain I or domain of nature and social awareness: physiotherapy educators could empower students to make more complex and systematic decisions, such as in planning and co-designing projects with the community, locally or transnationally. Domain II or domain of abstraction: educators must seek the analysis of complex environmental concepts from a transdisciplinary perspective, their relationships and theoretical bases, as well as innovative positions to address environmental challenges. Domain III or domain of ethics: physiotherapy educators could strengthen ethical thinking promoting a critical reflection on equity and climate justice. Domain IV or content domain: Educators should deepen specific knowledge and skills about planetary health and other related approaches, including the selection, the production of evidence or the rescue of data for direct service, research or advocacy.

Physiotherapy curriculum should also consider the Primary Action Areas of the Global Plan of Action on climate change and health (PAA1, PAA2 and PAA3). The crossing point at the center of the curriculum (D) supposes the dialogic intersection of the strategic lines of action in the face of current eco-social challenges and the dimensions of service learning in planetary health. It is here again that service-learning must be seen not only as a pedagogical mediator but also as a curriculum regenerator, sustained by its profound capacity to stimulate critical thinking and reflection in and on action (91).

Service-learning is appropriate for *empowering*, *strengthening*, and *implementing* transdisciplinary initiatives related to change mitigation or adaptation but also for other forms of relating education and environment. Since service-learning is based on partnerships between the community and the university, strengthening and sustaining those over time is essential to produce transformative inputs into the curriculum (92). This is facilitated by the institutionalization of service-learning, a multifaceted process aimed at promoting and normalizing service-learning in higher education. This process is usually initiated by key people who follow a bottom-up or top-down approach (93). In the first case, the process starts from individual projects that are transferred and expanded to other disciplines, requiring an institutional process to support them. In the second, less frequent, institutionalization is based on the impulse of the organization’s leaders, who promote policies for the optimal development of service-learning throughout the organization. To this end, institutions must implement policies and management processes aimed at training educators in this pedagogy, empowering students, but also facilitating, promoting, and recognizing service-learning (76–78).

The future challenge for many faculties and schools is the development of matchmaking tools where educational needs are integrated and balanced with service requirements framed in planetary health. This can be particularly relevant to connect higher education with the rural world, establishing new formats of co-creation and collaboration based on reciprocity. Digital tools are helpful to enable physiotherapy students to document and showcase the skills gained through service-learning, making these competencies more visible, transferable, and meaningful to employers in order to continue transformations at workplace level. Another challenge refers to quality management and assessment procedures that accompany curricular transformations in physiotherapy. Qualitative data based on students’ self-reflection and community partners feedback, should be particularly considered for this regeneration process.

By promoting service-learning, physiotherapy schools and faculties could be seen as living laboratories or communities of practice, proposing planetary health solutions. But there are no magic recipes here. It will be necessary to delve into the purpose of education and the uncertainty of everyday life, while still instilling hope in the current challenges. This will require a critical analysis of processes and products, avoiding sporadic experiences with no real meaning or purpose. The establishment of a community of practice in service-learning would facilitate mentoring, as well as the development of transdisciplinary projects with students and scholars from other disciplines Finally, the improvement of physiotherapy education, research, and society’s commitment to planetary health can be promoted through quality assurance procedures that recognize social motivations for cooperation, as is the case with service-learning projects (94).

In a way of reading the future, the challenge of regenerating higher education toward sustainability and planetary health will be permanent, because that is not a destination where once people arrive, they can sit back and relax. It will require continuous experimentation, learning, reflection, connection, questioning, and recalibration. This must be observed through the lens of real life, that is, from uncertainty, complexity, and ambiguity, taking risks, accepting failures, and promoting a hopeful vision of the future (95–97). Because this affects us all. Physiotherapists included.

## References

[1] Abrandt M. Learning physiotherapy: the impact of formal education and professional experience. Linkoping Stud Educ Psychol. (1997) 50:1–169.

[2] Shumba TW Tekian A. Competencies of undergraduate physiotherapy education: a scoping review. S Afr J Physiother. (2024) 80:a1879. doi: 10.4102/sajp.v80i1.1879, PMID: 38322654 PMC10839158

[3] Paz-Lourido B. Home physiotherapy: the relevance of social determinants of health in the development of physiotherapy in the home environment In: Saltikov B Lourido P, editors. Physical therapy perspectives in the 21st century - challenges and possibilities. London: Tech Books (2012). 197–218.

[4] Martiáñez-Ramírez NL Pineda-Galán C Rodríguez-Bailón M Romero-Galisteo PR. Competence assessment rubric in the physiotherapy practicum. PLoS One. (2022) 17:e0264120. doi: 10.1371/journal.pone.0264120 35213586 PMC8880643

[5] Gibson BE Nixon AA Nicholls DA. Critical reflections on the physiotherapy profession in Canada. Physiother Can. (2010) 62:98–100. doi: 10.3138/physio.62.2.98 21359039 PMC2871015

[6] Narain S Mathye D. Do physiotherapists have a role to play in the sustainable development goals? A qualitative exploration. S Afr J Physiother. (2019) 75:1–9. doi: 10.4102/sajp.v75i1.466 PMC6494920 31061910

[7] Palstam A Andersson M Lange E Grenholm A. A call to include a perspective of sustainable development in physical therapy research. Phys Ther. (2020) 36:279–383. doi: 10.1093/ptj/pzaa228, PMID: 33382399 PMC7970626

[8] Palstam A Sehdev S Barna S Andersson M Liebenberg N. Sustainability in physiotherapy and rehabilitation. Orthop Traumatol. (2022) 36:279–83. doi: 10.1016/j.mporth.2022.07.005

[9] Swärdh E Maric F. From knowledge to action: fostering advocacy skills for planetary health in physical therapy. Phys Ther. (2024) 104:pzae130. doi: 10.1093/ptj/pzae130, PMID: 39239843 PMC11530356

[10] Li LSK Fryer CE Chi L Boucaut R. Physiotherapy and planetary health: a scoping review. Eur J Phys. (2024) 27. doi: 10.1080/21679169.2024.2323729

[11] Conceição P. (2020). Human development report 2020. The next frontier. Human development and the Anthropocene. 1–11. Available online at: https://hdr.undp.org/system/files/documents/hdr2020.pdf [Accessed November 8, 2024].

[12] Whitmee S Haines A Beyrer C Boltz F Capon AG de Souza Dias BF . Safeguarding human health in the Anthropocene epoch: report of the Rockefeller Foundation–lancet commission on planetary health. Lancet. (2015) 386:1973–2028. doi: 10.1016/s0140-6736(15)60901-1, PMID: 26188744

[13] Henrique KP Tschakert P du Bourgault Coudray C Horwitz P Krueger KDC Wheeler AJ. Navigating loss and value trade-offs in a changing climate. Clim Risk Manag. (2022) 35:100405. doi: 10.1016/j.crm.2022.100405

[14] Obura DO. Achieving a nature- and people-positive future. One Earth. (2022) 6:105–17. doi: 10.1016/j.oneear.2022.11.013

[15] Maric F Nicholls DA. Environmental physiotherapy and the case for multispecies justice in planetary health. Physiother Theory Pract. (2022) 38:2295–306. doi: 10.1080/09593985.2021.1964659, PMID: 34365892

[16] Paz-Lourido B. Service-learning ¿A methodology to consider in physiotherapy in higher education? Fisioterapia. (2017) 39:227–8. doi: 10.1016/j.ft.2017.09.005

[17] World Health Organization. (2025). Climate change and health. Executive board EB156/25. Draft global action plan on climate change and health. Available online at: https://apps.who.int/gb/ebwha/pdf_files/EB156/B156_25-en.pdf [Accessed January 31, 2025].

[18] Parums DV. A review of the increasing global impact of climate change on human health and approaches to medical preparedness. Med Sci Monit. (2024) 30:e945763. doi: 10.12659/msm.945763, PMID: 38988000 PMC11302257

[19] Mazumder H Hossain MM. Climate change education for health-care professionals: crucial gaps in low-income and middle-income countries. Lancet Planetary Health. (2024) 8:e216. doi: 10.1016/s2542-5196(24)00010-x, PMID: 38580422

[20] Neta G Pan W Ebi K Buss DF Castranio T Lowe R . Advancing climate change health adaptation through implementation science. Lancet Planetary Health. (2022) 6:e909–18. doi: 10.1016/s2542-5196(22)00199-1, PMID: 36370729 PMC9669460

[21] Pfenning-Butterworth A Buckley LB Drake JM Farner JE Farrell MJ Gehman ALM . Interconnecting global threats: climate change, biodiversity loss, and infectious diseases. Lancet Planetary Health. (2024) 8:e270–83. doi: 10.1016/s2542-5196(24)00021-4, PMID: 38580428 PMC11090248

[22] Iyer HS DeVille NV Stoddard O Cole J Myers MS Li H . Sustaining planetary health through systems thinking: public health's critical role. SSM - Population Health. (2021) 15:2021. doi: 10.1016/j.ssmph.2021.100844 PMC8213960 34179331

[23] Planetary Health Alliance. (2024). Kuala Lumpur call to action on planetary health. Available online at: https://www.planetaryhealthalliance.org/kuala-lumpur-call-to-action. [Accessed November 22, 2024].

[24] Kirwan M Bhatti AJ Pacey V Gray K Dean CM. Overcoming silos: a sustainable and innovative approach to curriculum development. Educ Sci. (2022) 12:375. doi: 10.3390/educsci12060375

[25] Freire. Pedagogy of hope: Reliving pedagogy of the oppressed. London: Bloomsbury Academic (2021).

[26] Rockström J. (2015). Bounding the planetary future: why we need a great transition. Available online at: https://www.greattransition.org/images/GTI_publications/Rockstrom-Bounding_the_Planetary_Future.pdf (accessed December 6, 2024).

[27] Adefila A Chen YF Chao CM Oyinlola M Anafi F. Developing transformative pedagogies for transdisciplinary education – resources and competencies students need. Innov Educ Teach Int. (2022) 60:476–87. doi: 10.1080/14703297.2022.2062032

[28] Schneider F Llanque-Zonta A Andriamihaja OR Andriatsitohaina RNN Tun AM Boniface K . How context affects transdisciplinary research: insights from Asia, Africa and Latin America. Sustain Sci. (2022) 17:2331–45. doi: 10.1007/s11625-022-01201-3, PMID: 36439030 PMC9684244

[29] Mallini KC. Leadership development in physical therapy: moving toward a community of transformative practitioners. Theses Dissertations-Educ Leadership Stud. (2019) 25:1–212.

[30] Talukder B Ganguli N Choi E Tofighi M Vanloon GW Orbinski J. Exploring the nexus: comparing and aligning planetary health, one health, and ecohealth. Glob Transit. (2024) 6:66–75. doi: 10.1016/j.glt.2023.12.002

[31] Halonen JI. A call for urgent action to safeguard our planet and our health in line with the Helsinki declaration. Environ Res. (2020) 193:110600. doi: 10.1016/j.envres.2020.110600 33307082

[32] Destoumieux-Garzón D Mavingui O Boetsch G Boissier J Darriet F Duboz P . The one health concept: 10 years old and a long road ahead. Front Vet Sci. (2018) 5. doi: 10.3389/fvets.2018.00014 PMC5816263 29484301

[33] De Paula N. Planetary health diplomacy: a call to action. Lancet Planetary Health. (2021) 5:e8–9. doi: 10.1016/s2542-5196(20)30300-4, PMID: 33421411

[34] Mackenzie JS Jeggo M. The one health approach—why is it so important? Tropical Med Infectious Dis. (2019) 4:88. doi: 10.3390/tropicalmed4020088, PMID: 31159338 PMC6630404

[35] Lerner H Berg C. A comparison of three holistic approaches to health: one health, EcoHealth, and planetary health. Front Vet Sci. (2017) 4. doi: 10.3389/fvets.2017.00163, PMID: 29085825 PMC5649127

[36] Roger F Roger F Caron A Morand S Pedrono M Garine-Wichatitsky M. One health and ecohealth: the same wine in different bottles? Infect Ecol Epidemiol. (2016) 6:30978. doi: 10.3402/iee.v6.30978, PMID: 26899935 PMC4761681

[37] Van Herten J Bovenkerk B Verweij M. One health as a moral dilemma: towards a socially responsible zoonotic disease control. Zoonoses Public Health. (2019) 66:26–34. doi: 10.1111/zph.12536, PMID: 30390380 PMC7379490

[38] De Castañeda RR Villers J Faerron CA Eslanloo E de Paula N Machalaba C . One health and planetary health research: leveraging differences to grow together. Lancet Planet Health. (2023) 7:e109–11. doi: 10.1016/s2542-5196(23)00002-5 36754465 PMC9901939

[39] Olea-Popelka F Redvers N Stranges S. Public health, one health, and planetary health: what is next? Eur J Pub Health. (2024) 35:3–5. doi: 10.1093/eurpub/ckae149 PMC11832140 39378415

[40] Ridder-Symoens H. A history of the university in Europe: Volume 1, universities in the middle ages. Cambridge: Cambridge University Press (1992).

[41] United Nations Educational, Scientific and Cultural organization (2024). Higher education. Available online at: https://www.unesco.org/en/higher-education [Accessed October 1, 2024].

[42] United Nations Educational, Scientific and Cultural organization (2009). World conference on higher education. The new dynamics of higher educatin and research for societal change and development. Available online at: https://unesdoc.unesco.org/ark:/48223/pf0000183277_spa [Accessed May 23, 2024].

[43] Filho WL Leal Filho W Sima M Sharifi A Luetz JM Salvia AL . Handling climate change education at universities: an overview. Environ Sci Eur. (2021) 33:109. doi: 10.1186/s12302-021-00552-5, PMID: 34603904 PMC8475314

[44] Planetary Health Alliance (2024). São Paulo declaration on planetary health. Available online at: https://www.planetaryhealthalliance.org/sao-paulo-declaration. [Accessed September 12, 2024].

[45] United Nations (2015). “Transforming our world: the 2030 agenda for sustainable development”, resolution adopted by the general assembly. Available online at: www.sus-tainabledevelopment.un.org/post2015/transformingourworld (accessed September 10, 2024).

[46] Mulà I Tilbury D Ryan A Mader M Dlouhá J Mader C . Catalysing change in higher education for sustainable development: a review of professional development initiatives for university educators. Int J Sustain High Educ. (2017) 18:798–820. doi: 10.1108/IJSHE-03-2017-0043

[47] Australia SDSN Zealand New Edition Pacific (2017). Getting started with the SDGs in universities: A guide for universities, higher education institutions, and the aca-demic sector. 2017. Available online at: www.ap-unsdsn.org/wp-con-ent/uploads/2017/08/University-SDG-Guide_web.pdf (accessed October 8, 2024).

[48] Reckien D Lwasa S Satterthwaite D McEvoy D Creutzig F Montgomery M. Equity, environmental justice, and urban climate change In: Rosenzweig C Solecki W Romero-Lankao P Mehrotra S Dhakal S Ali Ibrahim S, editors. Climate change and cities: Second assessment report of the urban climate change research network. New York: Cambridge University Press (2018). 173–224.

[49] London J. Environmental justice and regional political ecology converge in the other California. J Polit Ecol. (2016) 23:147–158. doi: 10.2458/v23i1.20186

[50] ESCAP. (2015). Time for equality. The role of social protection in reducing inequalities in Asia and the Pacific. United Nations. Available online at: https://www.unescap.org/sites/default/files/SDD%20Time%20for%20Equality%20report_final.pdf (accessed June 30, 2024).

[51] Leibowitz B. Cognitive justice and the higher education curriculum. J Educ. (2017) 68:93–111. doi: 10.17159/2520-9868/i68a03

[52] Bhambra GK Newell P. More than a metaphor: ‘climate colonialism’ in perspective. Global Soc Challenges J. (2022) 2:179–87. doi: 10.1332/eiem6688

[53] Mbiekop F. Okoli N. International Development Research Center (2023) Gender, unpaid care and social protection: policy priorities for west and Central Africa, expert group meeting Available online at: https://www.unwomen.org/sites/default/files/2024-01/gender_unpaid:care_and_social_protection_policy_priorities_west_and_central_africa.pdf (accessed November 3, 2024).

[54] Manolas E. Short stories and climate change: an application of Kolb’s experiential learning model. Climate Change Manag. (2017) 2:37–46. doi: 10.1007/978-3-319-70066-3_3

[55] Van den Berg B Poldner K Sjoer E Wals A. Practices, drivers and barriers of an emerging regenerative higher education in the Netherlands: a podcast-based inquiry. Sustain For. (2022) 14:9138. doi: 10.3390/su14159138

[56] Wessels KR Grünwald L. Fulfilling the regenerative potential of higher education: a collaborative auto-ethnography. Educ Sci. (2023) 13:1037. doi: 10.3390/educsci13101037

[57] Segalàs J Tejedor G. The role of transdisciplinarity in research and education for sustainable development. Res Innovation Educ Sustain Develop. (2016):197–210.

[58] Brand G Wise S Bedi G Kickett R. Embedding indigenous knowledges and voices in planetary health education. Lancet Planetary Health. (2023) 7:e97–e102. doi: 10.1016/s2542-5196(22)00308-4, PMID: 36608956

[59] White PJ Ardoin NM Eames C Monroe M. Agency in the Anthropocene: education for planetary health. Lancet Planet Health. (2024) 8:e117. doi: 10.1016/s2542-5196(23)00271-1, PMID: 38331528

[60] Arora R Spikes E Waxman-Lee C Arora R. Platforming youth voices in planetary health leadership and advocacy: an untapped reservoir for changemaking. Lancet Planet Health. (2022) 6:e78–80. doi: 10.1016/s2542-5196(21)00356-9, PMID: 35150629

[61] European Youth Energy Forum (2022). The role of youth in the future of the European energy transition Available online at: https://youthenergy.eu/wp-content/uploads/2022/12/Role-Youth-European-Energy-Transition-Position-paper.pdf (accessed June 5, 2024).

[62] World Physiotherapy. Policy statement. Climate change and health. London, UK: World Physiotherapy (2023).

[63] World Physiotherapy (2022). Ethical principles and the responsibilities of physiotherapists and member organizations Available online at: https://world.physio/sites/default/files/2022-03/PS-2022-Ethical_responsibilities_principles_Eng.pdf (accessed July 17, 2024).

[64] Nguyen P-Y Astell-Burt T Rahimi-Ardabili H Feng X. Effect of nature prescriptions on cardiometabolic and mental health, and physical activity: a systematic review. Lancet Planetary Health. (2023) 7:e313–28. doi: 10.1016/s2542-5196(23)00025-6, PMID: 37019572

[65] Shanahan D Astell–Burt T Barber E Brymer E Cox D Dean J . Nature–based interventions for improving health and wellbeing: the purpose, the people and the outcomes. Sports. (2019) 7:141. doi: 10.3390/sports7060141, PMID: 31185675 PMC6628071

[66] Astell-Burt T Pappas E Redfern J Feng X. Nature prescriptions for community and planetary health: unrealised potential to improve compliance and outcomes in physiotherapy. J Phys. (2022) 68:151–2. doi: 10.1016/j.jphys.2022.05.016, PMID: 35753967

[67] Ratima M Martin D Castleden H Delormier T. Indigenous voices and knowledge systems – promoting planetary health, health equity, and sustainable development now and for future generations. Glob Health Promot. (2019) 26:3–5. doi: 10.1177/1757975919838487, PMID: 30964406

[68] Kronenberg J Andersson E Elmqvist T Łaszkiewicz E Xue J Khmara Y. Cities, planetary boundaries, and degrowth. Lancet Planetary Health. (2024) 8:e234–41. doi: 10.1016/S2542-5196(24)00025-1, PMID: 38580425

[69] Kniffin LE Clayton PH Camo-Biogradlija J Price MF Bringle RG Botkin HM. Using a critical reflection framework to deepen community-campus relationships and partnerships: a multi-institutional mixed-methods study. Int J Res Service-Learn Community Engagement. (2023) 11:1-18.

[70] Dewey J. Experience and education. New York: MacMillan (1938).

[71] Aramburuzabala P Cerrillo R. Service-learning as an approach to educating for sustainable development. Sustain For. (2023) 15:11231. doi: 10.3390/su151411231

[72] Coleman K Murdoch J Rayback S Seidl A Wallin K. Students’ understanding of sustainability and climate change across linked service-learning courses. J Geosci Educ. (2017) 65:158–67. doi: 10.5408/16-168.1

[73] Mateus C Potito A Curley M. Engaging secondary school students in climate data rescue through service-learning partnerships. Weather. (2020) 76:113–8. doi: 10.1002/wea.3841

[74] Davis AY Green SGK Heppard A. An intentionally designed sustainability course: integrating service-learning and community engagement, journal of sustainability education. J Sustain Educ. (2024) 29

[75] Kalafatis SE Neosh J Libarkin JC Whyte KP Caldwell C. Experiential learning processes informing climate change decision support. Weather Clim Soc. (2019) 11:681–94. doi: 10.1175/wcas-d-19-0002.1

[76] Bringle R Hatcher J. Institutionalization of service-learning in higher education. J High Educ. (2000) 71:273–90. doi: 10.1080/00221546.2000.11780823

[77] Furco A. Institutionalizing service-learning in higher education. J Public Aff. (2002) 6:39–47.

[78] Ribeiro A Aramburuzabala P Paz-Lourido B. Guidelines for the institutionalization of service-learning in European higher education. Madrid: European Association of Service-Learning in Higher Education (2021).

[79] Stater KJ Fotheringham E. Mechanisms for institutionalizing service-learning and community partner outcomes. J High Educ Outreach Engagem. (2009) 13:7–30.

[80] Foo R. The role of physiotherapy in climate change mitigation. Physiotherapy. (2015) 102:e5. doi: 10.1016/j.physio.2015.10.009 27137087

[81] Rowland J. Importance of physiotherapists in promoting sustainable community-based health programmes to improve long-term health outcomes. N Z J Physiother. (2016) 44:73–4. doi: 10.15619/nzjp/44.2.01

[82] Hancock T. Population health promotion 2.0: an eco-social approach to public health in the Anthropocene. Can J Public Health. (2015) 106:e252–5. doi: 10.17269/cjph.106.5161, PMID: 26285199 PMC6972308

[83] Faerron AF Aguirre A Astle B Barros E Baylese B Chimbarif M. A framework to guide planetary health education. Lancet Planet Health. (2021) 5:e253–5. doi: 10.1016/s2542-5196(21)00110-8 33894134

[84] Stanhope J Maric F Rothmore P Weinstein P. Physiotherapy and ecosystem services: improving the health of our patients, the population, and the environment. Physiother Theory Pract. (2021) 39:227–40. doi: 10.1080/09593985.2021.2015814, PMID: 34904927

[85] Bach AJE Cunningham SJK Morris NR Xu Z Rutherford S Binnewies S . Experimental research in environmentally induced hyperthermic older persons: a systematic quantitative literature review mapping the available evidence. Temperature. (2023) 11:4–26. doi: 10.1080/23328940.2023.2242062, PMID: 38567267 PMC7615797

[86] Kolb AY Kolb DA. Experiential learning theory: a dynamic, holistic approach to management learning, education and development In: Amstrong SJ Fukami CV, editors. The SAGE handbook of management learning, education and development. Thousand Oaks, CA: SAGE (2009). 42–68.

[87] Roberts J. Experiencing sustainability: thinking deeper about experiential education in higher education. J Sustain Educ. (2013) 5:369–84.

[88] Pesqueux Y. Sustainable development: a vague and ambiguous “theory”. Soc Bus Rev. (2009) 4:231–45. doi: 10.1108/17465680910994227

[89] Redvers N Celidwen Y Schultz C Horn O Githaiga C Vera M . The determinants of planetary health: an indigenous consensus perspective. Lancet Planetary Health. (2022) 6:e156–63. doi: 10.1016/S2542-5196(21)00354-5, PMID: 35150624

[90] Prah E Bland S. Climate change and healthscapes: toward a postcolonial critique for planetary well-being. S Afr Rev Sociol. (2024) 54:448–63. doi: 10.1080/21528586.2024.2417172

[91] Kawai T. A theoretical framework on reflection in service learning: deepening reflection through identity development. Front Educ. (2021) 5:1–11. doi: 10.3389/feduc.2020.604997

[92] Bringle RG Clayton PH. Higher education: service-learning as pedagogy, partnership, institutional organization, and change strategy In: Tierney RJ Rizvi F Ercikan K, editors. International encyclopedia of education, vol. 8. Amsterdam: Elsevier (2023). 476–90.

[93] Tijsma G Urias E Zweekhorst M. Embedding engaged education through community service learning in HEI: a review. Educ Res. (2023) 65:143–69. doi: 10.1080/00131881.2023.2181202

[94] Paz-Lourido B (2024). More than words: an integrative approach to cooperation and quality assurance in higher education through service-learning Available online at: https://www.eua.eu/publications/conference-papers/more-than-words-an-integrative-approach-to-cooperation-and-quality-assurance-in-higher-education-through-service-learning.html (accessed October 23, 2024).

[95] Macintyre T Tilbury D Wals A. Education and learning for sustainable futures. Oxfordshire: Routledge (2024).

[96] Paz-Lourido B. El aprendizaje-servicio y la salud universitaria. Bilbao: Asociación de Aprendizaje-Servicio Universitario (2023).

[97] International Commission on the Futures of Education. Reimagining our futures together: A new social contract for education. París: UNESCO (2021).

[98] Myers S Frumkin H. Planetary health. Protecting nature to protect ourselves. Washington DC: Island Press (2020).

[99] International Classification of Functioning, Disability and Health (ICF). Checklist. Available at: https://www.who.int/publications/m/item/icf-checklist (accessed October 8, 2024).

[100] Furco A. “Service-Learning: A Balanced Approach to Experiential Education.” Expanding Boundaries: Serving and Learning. Washington DC: Corporation for National Service (1996) 2–6.

